# Strontium chloride improves bone mass by affecting the gut microbiota in young male rats

**DOI:** 10.3389/fendo.2023.1198475

**Published:** 2023-09-15

**Authors:** Xueyao Xi, Yanan Gao, Jiaqi Wang, Nan Zheng

**Affiliations:** ^1^ Key Laboratory of Quality & Safety Control for Milk and Dairy Products of Ministry of Agriculture and Rural Affairs, Institute of Animal Sciences, Chinese Academy of Agricultural Sciences, Beijing, China; ^2^ Laboratory of Quality and Safety Risk Assessment for Dairy Products of Ministry of Agriculture and Rural Affairs, Institute of Animal Sciences, Chinese Academy of Agricultural Sciences, Beijing, China; ^3^ State Key Laboratory of Animal Nutrition, Institute of Animal Sciences, Chinese Academy of Agricultural Sciences, Beijing, China

**Keywords:** strontium, bone quality, gut microbiota, bone mass accumulation, prevention concept

## Abstract

**Introduction:**

Bone mass accumulated in early adulthood is an important determinant of bone mass throughout the lifespan, and inadequate bone deposition may lead to associated skeletal diseases. Recent studies suggest that gut bacteria may be potential factors in boosting bone mass. Strontium (Sr) as a key bioactive element has been shown to improve bone quality, but the precise way that maintains the equilibrium of the gut microbiome and bone health is still not well understood.

**Methods:**

We explored the capacity of SrCl2 solutions of varying concentrations (0, 100, 200 and 400 mg/kg BW) on bone quality in 7-week-old male Wistar rats and attempted to elucidate the mechanism through gut microbes.

**Results:**

The results showed that in a Wistar rat model under normal growth conditions, serum Ca levels increased after Sr-treatment and showed a dose-dependent increase with Sr concentration. Three-point mechanics and Micro-CT results showed that Sr exposure enhanced bone biomechanical properties and improved bone microarchitecture. In addition, the osteoblast gene markers *BMP*, *BGP, RUNX2*, *OPG* and *ALP* mRNA levels were significantly increased to varying degrees after Sr treatment, and the osteoclast markers RANKL and TRAP were accompanied by varying degrees of reduction. These experimental results show that Sr improves bones from multiple angles. Further investigation of the microbial population revealed that the composition of the gut microbiome was changed due to Sr, with the abundance of 6 of the bacteria showing a different dose dependence with Sr concentration than the control group. To investigate whether alterations in bacterial flora were responsible for the effects of Sr on bone remodeling, a further pearson correlation analysis was done, 4 types of bacteria (*Ruminococcaceae_UCG-014*, *Lachnospiraceae_NK4A136_group*, *Alistipes* and *Weissella*) were deduced to be the primary contributors to Sr-relieved bone loss. Of these, we focused our analysis on the most firmly associated *Ruminococcaceae_UCG-014*.

**Discussion:**

To summarize, our current research explores changes in bone mass following Sr intervention in young individuals, and the connection between Sr-altered intestinal flora and potentially beneficial bacteria in the attenuation of bone loss. These discoveries underscore the importance of the “gut-bone” axis, contributing to an understanding of how Sr affects bone quality, and providing a fresh idea for bone mass accumulation in young individuals and thereby preventing disease due to acquired bone mass deficiency.

## 1 Introduction

Data from a survey report released by China’s National Health Commission in 2018 showed that the national low-bone mass population is huge, with the low-bone mass rate reaching 32.9% in people aged 40-49 years, and the low-bone mass rate in people aged 50 years or older accounting for a staggering 46.4% (1). Low bone mass may be an important cause of decreased skeletal muscle mass, increased severity of osteoarthritis, and increased susceptibility to osteoporotic fractures (2–5). It has been shown that bone mass formed after growth in early adulthood is a major determinant of bone mass in aging (6). Approximately 90% of bone mass is reached by the age of 18 years, and this early stage of development is sometimes even referred to as the “bone bank” (7), meaning that bone mass accumulation during adolescence may be important throughout the life cycle. Therefore, in order to better implement the health concept of “prevention> treatment”, an in-depth study of the mechanisms of bone metabolism and a timely understanding of bone health is essential to optimize bone development during adolescence and to avoid the serious consequences of orthopedic diseases that may result later in life.

The gut microbiota, as an important endogenous regulator, exerts a variety of physiological roles, such as mechanical, biological and immune barriers to host (8). Recent research has revealed that the gut microbiota and bone metabolism are tightly connected, and that imbalances in the gut microbiota can alter the alkalinity of the gut, affecting calcium and vitamin D absorption and thus bone mineralization (9–12). Sjögren et al. showed that germ-free mice exhibited higher trabecular bone mineral density (vBMD) and improved trabecular histomorphometric indices compared to conventionally reared (CONV-R) mice (13). Meanwhile, a colonization experiment found that a predominant increase in bone growth in chronically colonized mice (14). Mounting proof suggests that the gut microbiome is a key factor in controlling bone quality, and therefore, the gut microbiome could be a promising treatment for relieving bone loss.

Researchers have also been actively exploring exogenous substances in response to the bone loss problem, and in recent years a number of researchers have taken a keen interest in SrCl_2_. A study on 12-week-old male rats administered SrCl_2_ for 8 consecutive weeks found enhanced osteoblast activity and increased bone mass (15); another experiment using different concentrations of SrCl_2_ on osteoclasts showed a decrease in osteoclast formation and a decrease in the total amount of bone resorption pit formation (16). This suggests that Sr has a dual osteogenic effect, possibly stimulating osteoblasts and inhibiting osteoclast activity simultaneously (17). In addition to this, numerous studies have confirmed that Sr is a key bioactive element in regulating bone turnover and maintaining skeletal homeostasis by coordinating the balance between osteoblasts and osteoclasts (18–20).

**Figure 1:**
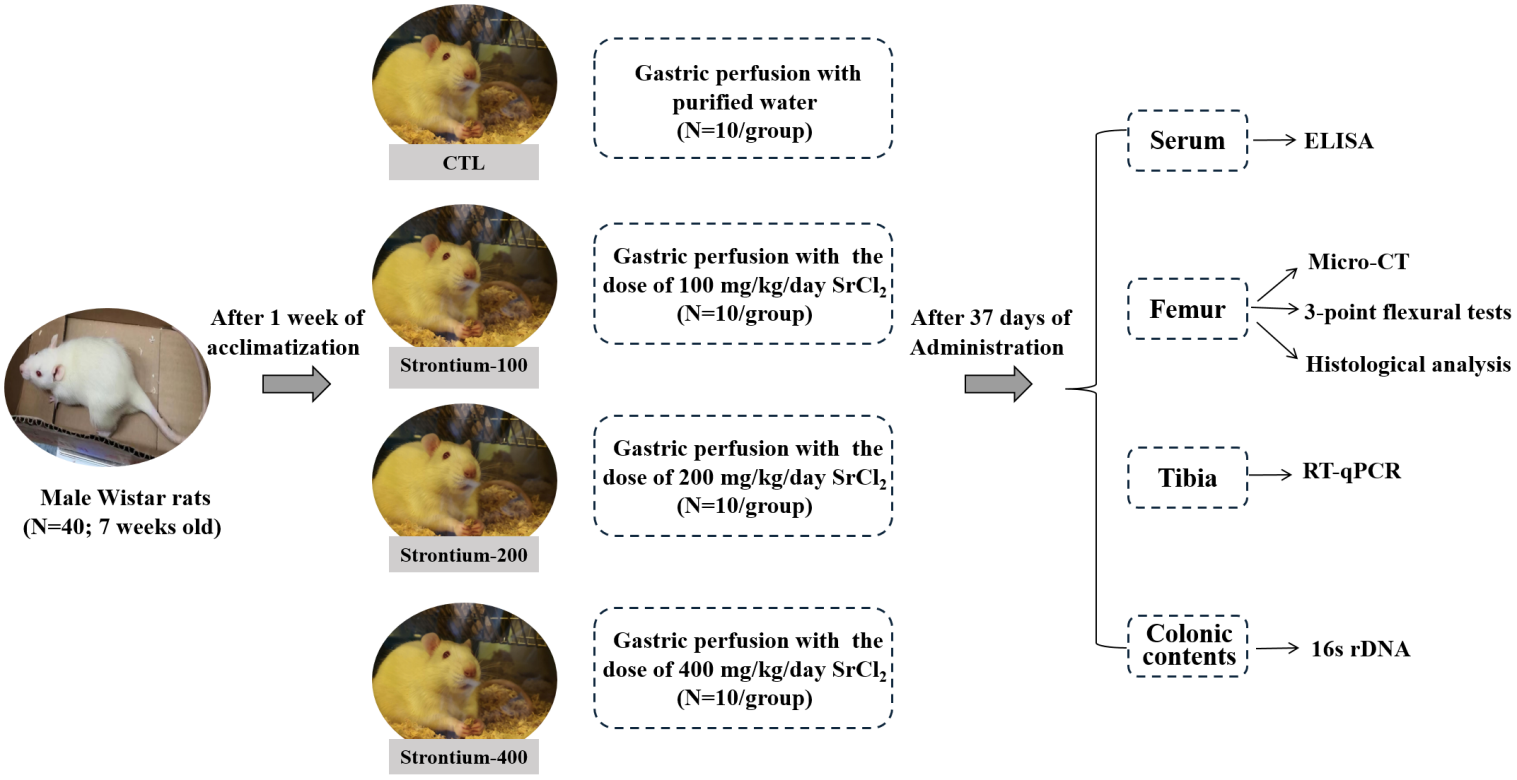
Experimental flow chart.

However, despite many experimental results demonstrating that Sr can improve bone quality and a large number of researchers discussing the relationship between gut microbiota and bone balance, no one seems to link the three together yet, and whether the gut microbiota can play a role in the improvement of bone quality by SrCl_2_ has not been reported and elucidated. Therefore, this export aimed to observe the effects of SrCl_2_ on the bones of adolescent male Wistar rats after 37 days of gavage using the intestinal flora as a medium and to assess relevant indices of bone in terms of histopathology and biotechnology (**Figure 1**).

## 2 Materials and methods

### 2.1 Materials and animals

#### 2.1.1 Chemicals and reagents

SrCl_2_ hexahydrate (SrCl_2_·6H_2_O, cat#AKSL-051059) was an analytical reagent (AR) and was purchased from Beijing Aikesailun Biotechnology Co., Ltd. The ELISA kits of procollagen type 1 N-terminal propeptide (PINP, cat: MM-20710R2), osteocalcin (OCN, cat: MM-0622R2), C-terminal cross-linked telopeptide of type 1 collagen (CTX, cat: MM-0931R2), parathyroid hormone (PTH, cat: MM-0631R2) and Vitamin D_3_ (cat: MM-0527R2) were bought from Jiangsu Meimian Industrial Co., Ltd. The HiPure Stool DNA kit was observed from Guangzhou Magen Biotechnology Co., Ltd.

#### 2.1.2 Animals and treatments

40 male Wistar rats (7 weeks old) were purchased from Beijing Vital River Co., Ltd. All of the rats participated in the study with unrestricted access to pure water and diet at 22 ± 3°C during the 12/12-hour light-dark cycle. The Committee on the Ethics of Animal Experiments of the Chinese Academy of Agriculture Sciences accepted the study’s procedure (permission number: IAS2022-146).

Following a week of acclimatization, the rats were arbitrarily divided into four groups. One group received (1) purified water (CTL group), while the other three received SrCl_2_·6H_2_O at doses of (2) 100 mg/kg BW (Sr-100 group), (3) 200 mg/kg BW (Sr-200 group), and (4) 400 mg/kg BW (Sr-400 group) daily by gavage for 37 days. Rats that had been fasting for 12 hours were weighed and anaesthetised at the conclusion of the experiment, serum was extracted and stored at -80°C. Subsequently, the connective and fatty tissues are removed to obtain femurs, tibias and colonic contents. The left femurs for Micro-CT scanning, histological assays and stored at -20°C. The right femurs for three-point mechanical experiments and stored at 4°C. The tibias for RT-qPCR experiments and the colonic contents for 16S rDNA amplicon sequencing and kept at -80°C. Samples are kept securely until further analysis.

### 2.2 Bone analysis

#### 2.2.1 Bone turnover markers of the serum

Serum calcium concentration was measured with an automatic biochemical analyzer (Rayto, Chemray 800). Serum indicators of bone turnover, PINP, OCN, CTX, PTH and Vitamin D3 were measured using ELISA kits with the manufacturers’protocols. Each assay included the testing of each sample in triplicate.

#### 2.2.2 Micro-computed tomography analysis

The Micro-CT (SkyScan 1176, Bruker) was used to scan the left femur with the following parameters: medium resolution 70KV, 200uA, voxel size 6.534165um and integration time 350ms. After scanning, the original images were reconstructed using the 3D reconstruction software NRecon (version V1.7.4.2) with the following parameters: Smoothing=5, Beamn-hardening=8, etc. Trabeculae in a 300-layer cross-section of the distal femur were defined as regions of interest (ROI), and the ROI were analysed using CT Analyser (version 1.20.3.0) after reconstruction of the images: trabecular number (Tb.N, 1/mm), bone mineral density (BMD, g/cm^3^), bone surface area (BS, mm^2^), bone volume/tissue volume (BV/TV, %), trabecular thickness (Tb.Th, mm) and structural model index (SMI, 1).

#### 2.2.3 Bone mechanical testing

The right femur was placed on two vertically parallel stands of the texture analyser (TAXT Plus, Stable Micro Systems) for the three-point bending test and loaded at a constant speed (24 mm/min). The maximum load (N), the breaking deformation (mm), the energy to ultimate load (mj), the strength at breaking point (N), the deformation at the maximum bending strength (mm) and the maximum energy before bending (mj) were recorded and calculated.

#### 2.2.4 Hematoxylin-Eosin staining

The fixed femur was decalcified in EDTA decalcification fluid, embedded and sliced. We stained the sections using hematoxylin and eosin, the nucleus is blue and the cytoplasm is red. We could see the boundary between the lacunae and bone matrix to evaluate the skeletal state.

#### 2.2.5 Tartrate-resistant acid phosphase staining

The fixed femur was decalcified in EDTA decalcification fluid, embedded and sliced. We incubated the sections with TRAP staining solution for 20 minutes. The cytoplasm of osteoclasts showed wine red and the nucleus showed light blue. When multinucleated cells are stained (≥3 nuclei), then they are defined as TRAP-positive.

#### 2.2.6 Gene expression assays

We used Trizol for the collection of total RNA from tibial bone marrow and detected the gene expression of osteogenic cytokines (*BMP, BGP, RUNX2, OPG, ALP, RANKL, TRAP*) on the Bio-Rad CFX96 system (Bio-Rad, Hercules, CA, USA). The *GAPDH* gene acted as the reference gene and the final ploidy changes were determined by the 2^-ΔΔCt^ method after normalization with the blank group. Each assay included the testing of each sample in triplicate and **Table 1** details the primer sequences for these genes.

**Table 1:** List of primers.

Primer	Direction	Sequence
GAPDH	Forward	GCAAGAGCACAAGAGGAAGAG
	Reverse	TCTACATGGCAACTGTGAGGA
BMP	Forward	AACAGCGGAAGCGTCTTAAGTCC
	Reverse	GGCATGGTTGGTGGAGTTCAGG
BGP	Forward	AAAGCCTTCATCTCCCACCG
	Reverse	AGCTCACACACCTCTCGTTG
RUNX2	Forward	CCACCACTCACTACCACACG
	Reverse	GGACGCTGACGAAGTACCAT
OPG	Forward	CACAGAGCAGCTCCGCATCTTG
	Reverse	AAGTGCTTGAGTGCGTACATCAGG
ALP	Forward	AGATGGATGAGGCCATCGGA
	Reverse	CCAAACGTGAAAACGTGGGA
RANKL	Forward	ATGATGGAAGGTTCGTGGCT
	Reverse	AAGAGGACAGACTGACTTTATGGG
TRAP	Forward	GACGCCAATGACAAGAGGT
	Reverse	AAACGCAAACGGTAATAAGG

BMP, bone morphogenetic protein; BGP, bone glaprotein; RUNX2, runt-related transcription factor 2; OPG, Osteoprotegerin; ALP, alkaline phosphatase; RANKL, receptor activator of nuclear factor-kB ligand; TRAP, tartrate resistant acid phosphatase.

### 2.3 Gut microbiota sequencing analysis

Five colon contents samples from each of the four groups were selected for DNA extraction and purification using the HiPure Stool DNA kit, followed by DNA quality assays to determine nucleic acid purity as well as integrity using a NanoDrop microspectrophotometer and agarose gel electrophoresis, respectively. Metagenomic DNA was used as a template for 16S ribosomal DNA (rDNA) amplification, with bacterial specific primers 341F (5’-CCTACGGGNGGCWGCAG-3’) and 806R (5’-GGACTACHVGGGTATCTAAT-3’) employed. After raw reads were obtained by sequencing on Illumina HiSeq using the MiSeq Reagent kit, low quality reads were filtered, total reads were combined, and clustering was performed to obtain OTUs. Bioinformatics analysis (α-diversity, β-diversity analysis, community composition and indicator species analysis) of the raw data was performed using various software including Qiime (version 1.9.1), Vegan package for the R language (version 2.5.3), R language ggplot2 package (version 2.2.1), and LEfSe (version 1.0). The raw data collected during the experiments are deposited in the NCBI Sequence Read Archive (SRA) database with the accession number: PRJNA986280. All sequencing and bioinformatics analysis were performed using the Omicsmart online platform (http://www.omicsmart.com).

### 2.4 Statistical analysis

Statistical analyses were performed using GraphPad Prism software (version 6.0). In the skeletal phenotype and gut microbiota sequencing analysis sections, we used one-way ANOVA; in the correlation analysis section, relationships between parameters were expressed as Pearson correlation coefficients and further analyses were performed using linear regression analysis. All results have at least 5 biological replicates (mean ± SEM). *, *P* <​ 0.05. **, *P* <​ 0.01. ***, *P* <​ 0.001.

## 3 Results

### 3.1 SrCl_2_ induces changes in bone conversion markers in the serum

**Figure 2:**
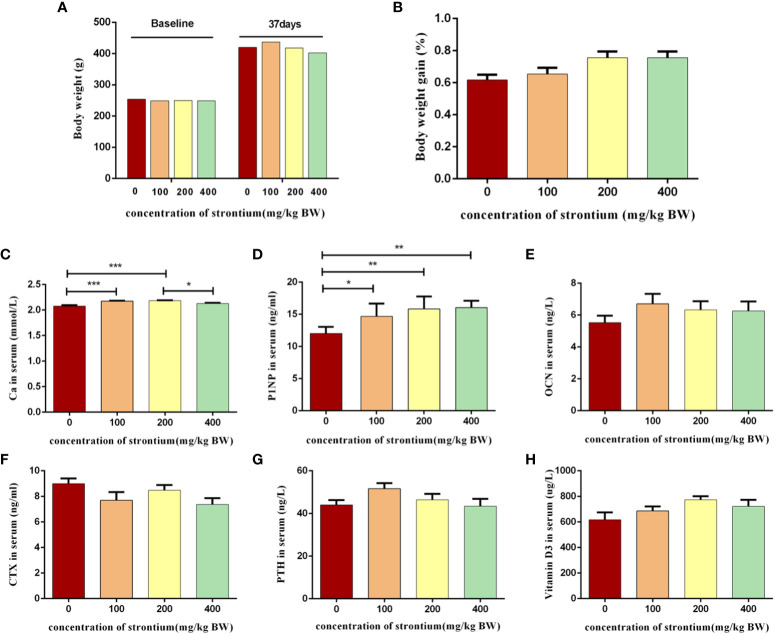
Effect of strontium treatment on growth and biochemical indicators of bone turnover in serum. **(A)** Initial and final body weight (n=8). **(B)** Body weight gain (n=8). **(C–H)** The level of Ca, PINP, OCN, CTX, PTH and Vitamin D_3_ in serum (n=6).

The animals’ survival rate which was treated with SrCl_2_ was comparable to that of the CTL group. The four experimental groups’ starting and ending body weights did not differ significantly from one another (*P*>0.05, **Figure 2A**). In terms of body weight gain, there was still no considerable difference (*P*>0.05, **Figure 2B**). This indicates that the rats’ consumption of SrCl_2_ did not cause negative effects such as aberrant weight growth or reduction.

Bone metabolism indicators are bone formation markers, bone resorption markers, etc., which were produced during bone transformation and their detection can represent osteoblast or osteoclast activity, reflecting the rate of bone formation or bone resorption. The Sr-infused group had significantly increased serum Ca levels than the CTL group (*P*<0.05, **Figure 2C**), indicating that Sr can affect the metabolism of bone minerals.​ Sr supplementation increased the bone formation metabolism indicators PINP levels than the CTL group (*P*<0.05, **Figure 2D**). Vitamin D_3_, OCN, PTH and CTX levels did not differ significantly between the groups (*P*>0.05, **Figures 2E–H**). The results of this experiment show that Sr can have a significant modulating effect on some bone turnover markers.

### 3.2 SrCl_2_ improves the bone microarchitecture of the femur

**Figure 3:**
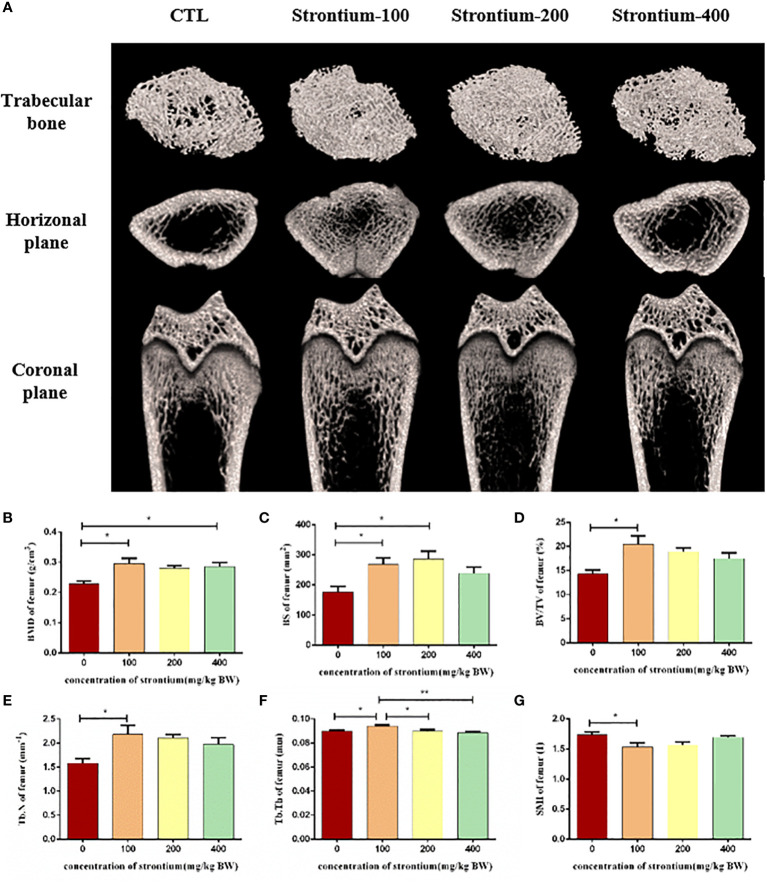
Effect of strontium treatment on bone microarchitecture. **(A)** Representative Micro-CT images. **(B–G)** Bone histomorphometry in BMD, BS, BV/TV, Tb. N, Tb.Th and SMI of the femur (n=5).

We used three-dimensional scans of the distal femur to observe changes in its internal microstructure following of SrCl_2_ treatment. The bone trabeculae in the distal femur of the CTL group were thinner and distributed more sparsely than those in the SrCl_2_ gavage group (**Figure 3A**). In comparison to the CTL group, the SrCl_2_ gavage group had a considerable rise in BMD, BS, BV/TV, Tb.N, Tb.Th and the shift were most pronounced in the Sr-100 group (*P*<0.05, **Figures 3B–F**). In contrast, the Sr-100 group had a significantly lower SMI than the CTL group (*P*<0.05, **Figure 3G**). The results of this experiment could mean that SrCl_2_ treatment has anti-skeletal condition deterioration potential.

### 3.3 SrCl_2_ improves the biomechanically relevant parameters of the femur

**Figure 4:**
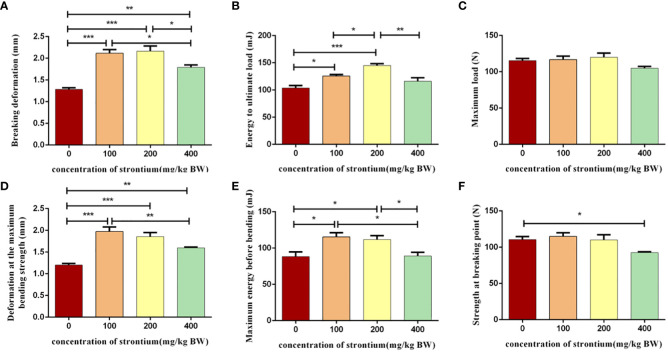
Effect of strontium treatment on bone biomechanics indicators. **(A–F)** Breaking deformation, energy to ultimate load, maximum load, deformation at the maximum bending strength, maximum energy before bending, strength at breaking point of the femur (n=6).

The femur was selected for a three-point mechanical experiment to investigate the effect of SrCl_2_ on the mechanical properties of the bone. Compression tests performed at the left femur showed a significant increase in breakage deformation, energy to ultimate load, deformation at the maximum bending strength and maximum energy before bending in the Sr-treated rats compared to the CTL group and this effect seems to be better in the Sr-100 and Sr-200 groups (*P*<0.05, **Figures 4A, B, D, E**). However, different to this situation the maximum load on the femur after treatment with Sr and the maximum energy before bending remained largely unchanged (*P*>0.05, **Figures 4C, F**). The test results suggest that using SrCl_2_ can improve the femur quality by enhancing the bone’s toughness, which is necessary for the bone to resist external impulses.

### 3.4 SrCl_2_ reduces the number of osteoclasts and changes the expression of related genes

**Figure 5:**
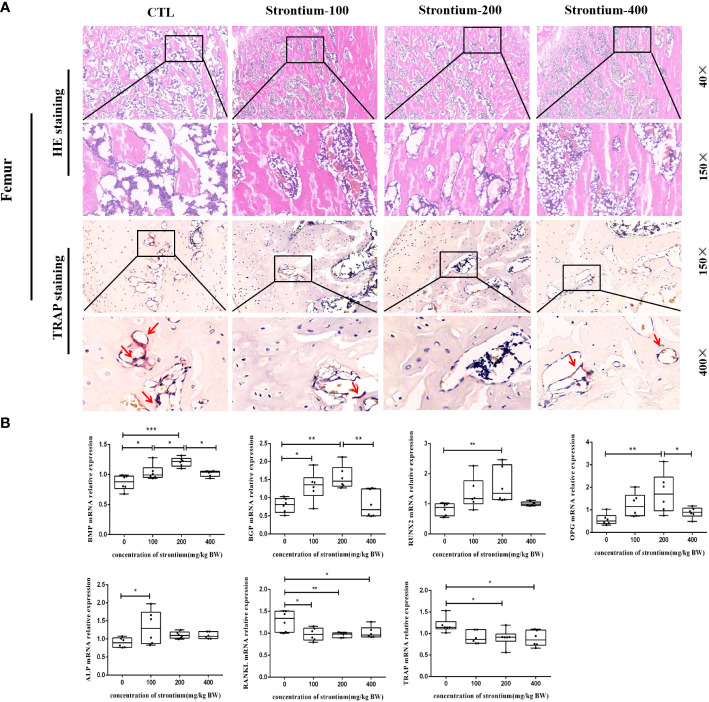
Effect of strontium treatment on bone histomorphology and bone-related genes indicators. **(A)** Representative images of femur tissue stained with HE (40×, 150× magnifications) and Trap (150×, 400× magnifications). **(B)** The relative mRNA expression of *BMP*, *BGP*, *RUNX2*, *OPG*, *ALP*, *RANKL, TRAP* (n=6).

The femur was removed for HE staining and Trap staining. The results of the HE-stained pathological sections showed that the morphology of the bone was compatible with the three-dimensional reconstructed images of Micro-CT. Following SrCl_2_ gavage, representative images of the Trap staining revealed a certain amount of osteoclast count reduction, the different doses of gavaged Sr showed different trends compared to the CTL group, with osteoclast formation significantly reduced in the Sr-200 group (**Figure 5A**).

Bone tissue formation is a complex and sequential process regulated by multiple growth factors. The mRNA levels of osteogenic conversion marker factors showed that the mRNA expression of *BMP* and *BGP* in the Sr-200 group was significantly increased and dose-dependent in the Sr-100 and Sr-200 groups, the expression levels of *RUNX2* and *OPG* were significantly higher in the Sr-200 group than in the CTL group, whereas the mRNA expression of *ALP* showed a significant increase in the Sr-100 group. Meanwhile, the osteoclast conversion marker factor *RANKL* showed a significant decrease in all groups, especially in the Sr-200 group, and the mRNA expression of *TRAP* showed a similar situation in the Sr-100 and Sr-200 groups (P<0.05) (**Figure 5B**). The results showed that SrCl_2_ could affect osteoclastogenesis, which could be corroborated by the expression of osteoclast marker mRNA, while the identification of osteogenic and osteoclast activity by bone conversion marker mRNA levels might be able to reflect bone development to some extent.

### 3.5 SrCl_2_ changes the gut microbiota structure

**Figure 6:**
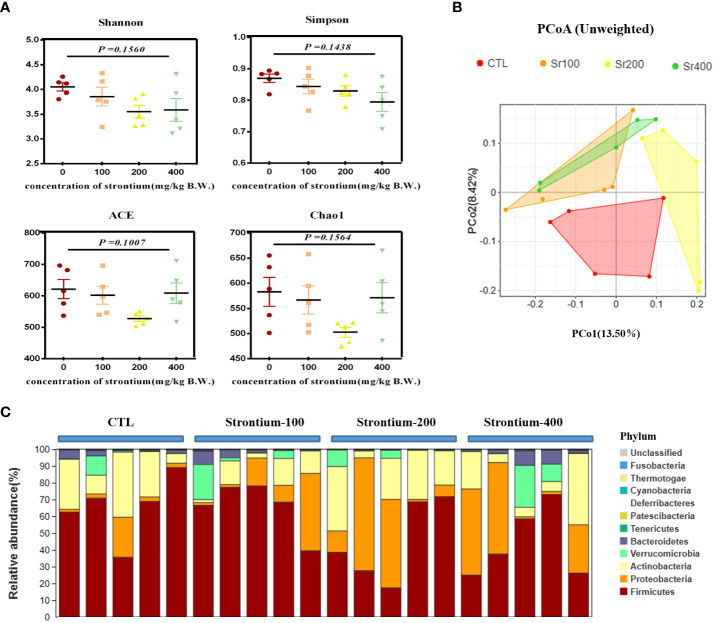
Effects of strontium treatment in reconstructing intestinal microbial ecosystem in Wistar rats. **(A)** Shannon, Simpson, ACE and Chao1 indexes. **(B)** Unweighted unifrac PCoA plot. **(C)** Composition of the bacterial community in colonic contents at the phylum level (n=5).

To assess whether the improvement in bone metabolism following Sr ingestion was related to the gut microbiota, further studies were conducted on the gut flora. The abundance and diversity of the intestinal flora as measured by the α-diversity indices (Shannon, Simpson, ACE and Chao1 indices) were somewhat diminished in the group exposed to SrCl_2_ (*P*>0.05, **Figure 6A**). The results of the unweighted_unifrac PCoA plot from the β-diversity analysis indicated that some data of the Sr-100 group and Sr-400 group have overlapping parts, while there was no overlapping with CTL group and Sr-200 group, this reminds us of the possible changes in the composition of the gut flora at 200 mg/kg of SrCl_2_ exposure (**Figure 6B**). At the phyla level, the top 10 dominant phylum were *Firmicute, Proteobacteria, Actinobacteria, Verrucomicrobia, Bacteroidetes, Tenericutes, Patescibacteria, Deferribacteres, Cyanobacteria* and *Thermotogae* (**Figure 6C**). Preliminarily, the available analyses demonstrate that the gut microbiota of Wistar rats is altered by the involvement of Sr, especially in the Sr-200 group.

### 3.6 SrCl_2_ altered the differential bacterial groups between the groups and composition in genus level

**Figure 7:**
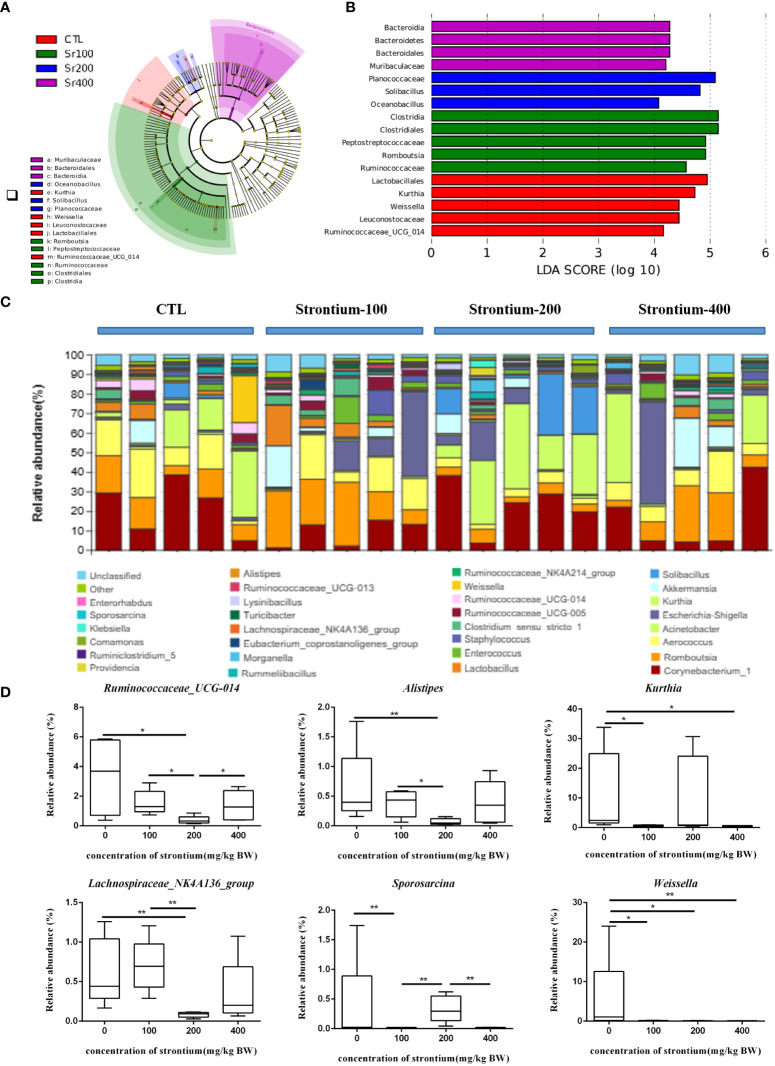
Effect of strontium treatment on gut microbiota alterations at the genus level in Wistar rats. **(A)** LEfSe taxonomic cladogram. **(B)** LDA score diagram. **(C)** Composition of the bacterial community in Colonic contents at the genus level. **(D)** *Ruminococcaceae_UCG-014, Alistipes, Kurthia, Lachnospiraceae_NK4A136_group, Sporosarcina* and *Weissella* at the genus level (n=5).

LEfSe analysis was applied to identify species that differed markedly in abundance among groups. The LDA distribution histogram suggests that the addition of Sr significantly increased the relative abundance of *Clostridia, Clostridiales and Peptostreptococcaceae* in the Sr-100 group. Moreover, the Sr-200 group increased the relative abundance of *Planococcaceae, Solibacillus* and *Oceanobacillus* and the Sr-400 group increased the relative abundance of *Bacteroidia, Bacteroidetes* and *Bacteroidales*. The results showed that the preponderant microbiota was different among the SrCl_2_-treated and CTL groups, the biomarker also changed in the Sr treatment due to different doses (**Figures 7A, B**). Collectively, these data imply that exposure to SrCl_2_ can regulate the composition of intestinal flora.

The average composition of the genus level shows the 30 genera with the highest abundance, 6 of which show dose-dependent variation in Sr concentrations different from the CTL group (**Figure 7C**). Compared to the CTL group, the relative abundances of *Ruminococcaceae_UCG-014, Alistipes* and *Lachnospiraceae_NK4A136_group* were lower in the Sr-100 and Sr-200 group, however, there was an increase by various amounts in the Sr-400 group. Compared with the CTL group, the relative abundances of *Kurthia* and *Sporosarcina* in the Sr-100 group and Sr-400 group were reduced to different degrees, however, these changes were not evident in the Sr-200 group. In addition, compared with the CTL group, the relative abundances of *Weissella* in the Sr exposure group was decreased to different degrees (*P*<0.05, **Figure 7D**). Further analysis showed that several biomarkers emerged after Sr exposure in Wistar rats and that some specific microflora also changed in abundance compared to the CTL group.

### 3.7 The correlation analysis between specific microbes and bone related indicators

**Figure 8:**
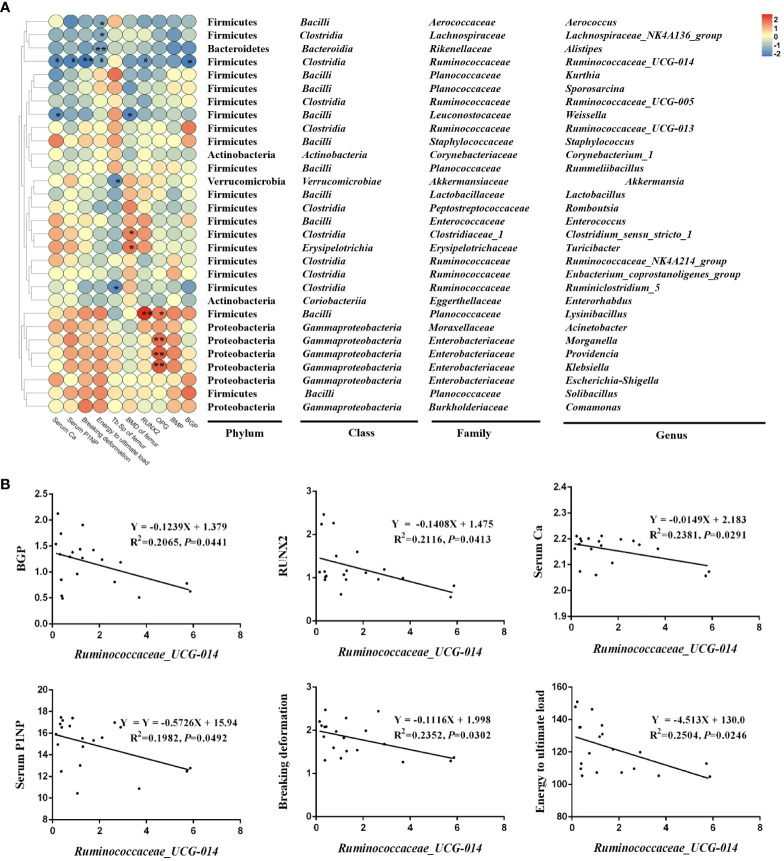
Correlation analysis of top 30 in abundance genera and other parameters. **(A)** Correlation heatmap of microbes and bone conversion-related indicators in the colon (top 18). **(B)** The simple linear regression of representative microbes, mainly *Ruminococcaceae_UCG-014* and skeletal-related indicators (n=5).

To examine the relationship between the composition of the intestinal microbiota and indicators related to bone turnover in Wistar rats, we performed a Pearson correlation analysis of the top 30 genera in terms of abundance and with bone conversion markers and found that several of these genera showed strong positive/negative relationships with certain specific bone conversion markers, they are *Aerococcus, Lachnospiraceae_NK4A136_group, Alistipes, Ruminococcaceae_UCG-014, Weissella, Akkermansia, Clostridium_sensu_stricto_1, Turicibacter, Ruminiclostridium_5, Lysinibacillus, Morganella, Providencia* and *Klebsiella* (**Figure 8A**). Among them, *Lachnospiraceae_NK4A136_group, Alistipes, Ruminococcaceae_UCG-014* and *Weissella* showed significant changes in their abundance with Sr treatment in the analysis of **Figure 7**. Therefore, we hypothesize that these four genera may be the key bacteria of Sr ameliorating bone quality.

*Ruminococcaceae_UCG-014* showed the broadest and strongest correlation with these bone indicators, so we have analyzed it in detail. In detail, *Ruminococcaceae_UCG-014*, a significantly down-regulated species in the Sr-gavaged group, was negatively correlated with *BGP* (R^2 ^= 0.2065, *P*=0.0441), *RUNX2* (R^2 ^= 0.2116, *P*=0.0413), Serum Ca (R^2 ^= 0.2381, *P*=0.0291), Serum PINP (R^2 = ^0.2504, *P*=0.0246), Breaking deformation (R^2 ^= 0.2352, *P*=0.0302) and Energy to ultimate load (R^2 ^= 0.2504, *P*=0.0246) (**Figure 8B**). These results suggested that the specie might play crucial roles in the bone conversion process, and thus is involved in the process of Sr alleviating bone loss.

## 4 Discussion

Numerous studies have confirmed the great potential of Sr in assisting osteogenesis, which can be achieved by simultaneously stimulating osteoblasts and inhibiting osteoclast activity to maintain bone mass (17–20). In addition to this, there is a growing body of research suggesting that dysbiosis of the gut microflora may trigger a reduction in bone mineral density and that there is a strong correlation between this and osteoporosis (21), there is an emerging consensus that the gut microbiota and its composition can have a significant function in regulating bone health (22). Although it is well documented that Sr can play a role in increasing bone mass (15, 23, 24), no studies have yet revealed whether the positive mechanisms are related to the gut flora and due to the high diversity of the gut microbiota, there is little knowledge about its structural composition and the impacts of specific flora on bone. Consequently, for this trial, we investigated the potential anti-osteoporosis action along the gut-bone axis of Sr administration after exposing 7-week-old intact male Wistar rats to various doses of SrCl_2_ (0, 100, 200 and 400 mg/kg BW) for 37 days. The evidence indicates that Sr regulates the gut microbiota to induce bone mass accumulation in adolescent male Wistar rats.

Compared to articles in which ovariectomized model was routinely performed (24), our results show no significant changes in body weight after treatment of normal healthy rats. This is probably due to the fact that we did not damage the model and thus did not cause a reduction in bone mass and body weight due to hormone deficiency and, secondly, to the fact that Sr does not harm on normal body development.

Bone transformation can be regulated by a specific bone remodeling process, and any imbalance in this specific process can cause bone disease (25). We found a striking increase in serum calcium levels in the Sr-100 and Sr-200 groups in this study. Previous studies have reported that reduced serum calcium levels can accelerate the bone conversion process (26), and adequate intestinal absorption of calcium is essential to reduce bone turnover (27). Our experimental results suggest that Sr may be responsible for reducing bone turnover and improving bone loss by increasing calcium intestinal absorption and serum calcium levels. A bone histomorphometric study of the lumbar spine in wild-type mice and transgenic mice showed that strontium ranelate (SrR) reversed the decrease in BV/TV, Tb.N caused by *Runx2* overexpressio (28). Prior investigation has demonstrated that Sr reversed the inhibitory action of glucocorticoids on bone marrow MSC osteogenesis and upregulated the expression of *RUNX2, ALP* and *BGP*, which is consistent with the results of this experiment (29).

In the present experiment, Sr showed a similar trend of improvement to previous studies, we also observed a more dense trabecular structure in the Micro-CT images, and the HE staining further confirmed our idea that Sr improves bone microarchitecture. The mechanical properties of bone correspond to bone mass, mineral quality, intrinsic tissue quality and integration of bone structure. Therefore, measuring the mechanical properties of bone is certainly the best way to assess changes in bone fragility after long-term treatment (30). The results of a study using 6-7 weeks old intact Fischer 344 rats as experimental animals showed a dose-dependent increase in bone strength and bone mass in the middle femur after strontium ranelate treatment, and similar to our experiments, the breaking deformation and energy to ultimate load of the rats were markedly improved in a dose-dependent manner. This increase in mechanical properties may be associated with improved microarchitecture, also showing an increase in Tb. N, Tb. Th, BV/TV, etc (31). Interestingly, another experiment also selected intact female Fischer rats aged 6–7 weeks old, which to further elucidate the relative contribution of structural and intrinsic tissue properties to the change in bone strength caused by strontium ranelate (32), it also inspired us to analyze the mechanism of Sr on the bone from more angles in subsequent experiments. It is worth mentioning that strontium ranelate used to be widely favored by patients as an anti-osteoporosis drug, but it has gradually withdrawn from the clinic in recent years, which may be due to the limitations of its application. Studies have reported that this drug use may be associated with cardiovascular risk characteristics (33). At the same time, strontium ranelate, as an unnatural synthetic compound, may be far less safe than natural Sr salts. Therefore, in the future, the direction we need to focus our exploration may center on how to produce Sr-rich foods that are safer and more efficiently absorbed, so that a person can supplement this beneficial element through a normal diet.

To further elucidate the relationship between changes in skeletal parameters and gut flora after Sr exposure, further studies were done, including 16S rDNA sequencing of colonic contents of rats and correlation analysis of specific gut microbes with bone-related markers. Firstly, the α diversity index results showed that Sr exposure did not affect the species diversity of the normal rat gut microbiota. The β diversity analysis of PCoA plots showed that the microbial communities of rats in the Sr-200 group exhibited distinctly different clusters from other instillation groups, suggesting that the community composition of Sr-200 group may have changed. Notably, results of selected phenotypic experiments on bones were also shown to be optimal in the Sr-200 group. We analyzed the colonies at the genus level and found by screening that *Ruminococcaceae_UCG-014, Alistipes, Kurthia, Lachnospiraceae_NK4A136_group, Sporosarcina* and *Weissella* were the specific bacteria whose abundance changed distinctly contrast with the CTL group. Correlation study was carried out to look into the target (s) of each bacterium among the prospective microorganisms. The results showed that *Ruminococcaceae_UCG-014, Alistipes, Lachnospiraceae_NK4A136_group* and *Weissella* demonstrated a significant link with skeletal-related indicators.

*Ruminococcaceae_UCG-014* and bone-related markers showed the broadest correlation, with their abundance correlating negatively with gene expression (*BGP, RUNX2*), serum bone turnover markers (Ca, PINP), biomechanical results (breaking deformation, energy to ultimate load) (*P*<​0.05). It is significant that in this experiment, the CTL group had the highest concentration of *Ruminococcaceae UCG-014*, but the involvement of Sr changed this situation, most significantly in the Sr-200 group (*P*<​0.05). In an experiment with dexamethasone-induced osteoporosis in rats, *Ruminococcaceae UCG-014* displayed a negative connection with bone-related parameters such as BMD, BV/TV and Tb.​N (34). We hypothesize that *Ruminococcaceae_UCG-014* may mediate some cellular and molecular signaling leading to the development of metabolic bone diseases.

In the present experiment, the abundance of *Alistipes* showed similar changes to that of *Ruminococcaceae_UCG-014*. A study using ovariectomy to establish an osteoporotic rat model to investigate the mechanisms of the intestinal bone axis found that the flora *Alistipes* could negatively affect bone metabolism (35), which is also in agreement with the results of this experiment. We suspect that *Alistipes* may be associated with elevated serum levels of inflammatory factors (36), which often induce cell differentiation and release of osteoclastogenic factors in osteoblasts (37). In addition to this, the *Lachnospiraceae_NK4A136_group* also showed the most significant changes in decreasing abundance when exposed to Sr at 200 mg/kg BW. Previous studies have reported that *Lachnospiraceae_NK4A136_group* is a bacterium that can produce short-chain fatty acids (38). *Weissella* has been less investigated in terms of pathology, with a previous study suggesting that *Weissella* caused a case of bacterial meningitis (37), and this paper is the first to identify a possible correlation between it and bone health. However, although *Weissella* has been reported less frequently in human infections, it still reminds us that attention should be paid to immunocompromised populations and could be explored further to gain a more accurate understanding of the impact of Sr-mediated changes in *Weissella* abundance on bone homeostasis.

Interestingly, but with less attention, current research has mainly focused on the regulation of bone metabolism by gut flora, but there seems to be no conclusive evidence on whether bone mass can influence gut flora. Starting from the time dimension, sampling at different time points to observe the dynamic changes of bone and intestinal microbiota may be a promising approach for exploring their causal relationship. Also of interest is the mode of action of strontium chloride. Some researchers believe that the use of drugs may affect the composition of the gut microbiota in different modes of action, with the first mode being that drugs cause the microbiota to migrate from other parts of the body to the gut. The second mode is that drugs directly affect bacterial growth by altering the intestinal microenvironment, which may be the main mode of action (39). We speculate that the effect of strontium chloride on the gut microbiota may be through the second mode, namely direct effect. The reason why microbial transfer factors are excluded is that, firstly, unlike tissues such as the oral cavity, bones have not the opportunity to come into contact with the outside world, thereby promoting microbial colonization and reproduction; secondly, unlike tissues such as the gut, bones cannot continuously obtain a diverse and nutrient rich matrix to maintain the survival of microorganisms.

## 5 Conclusions

**Figure 9:**
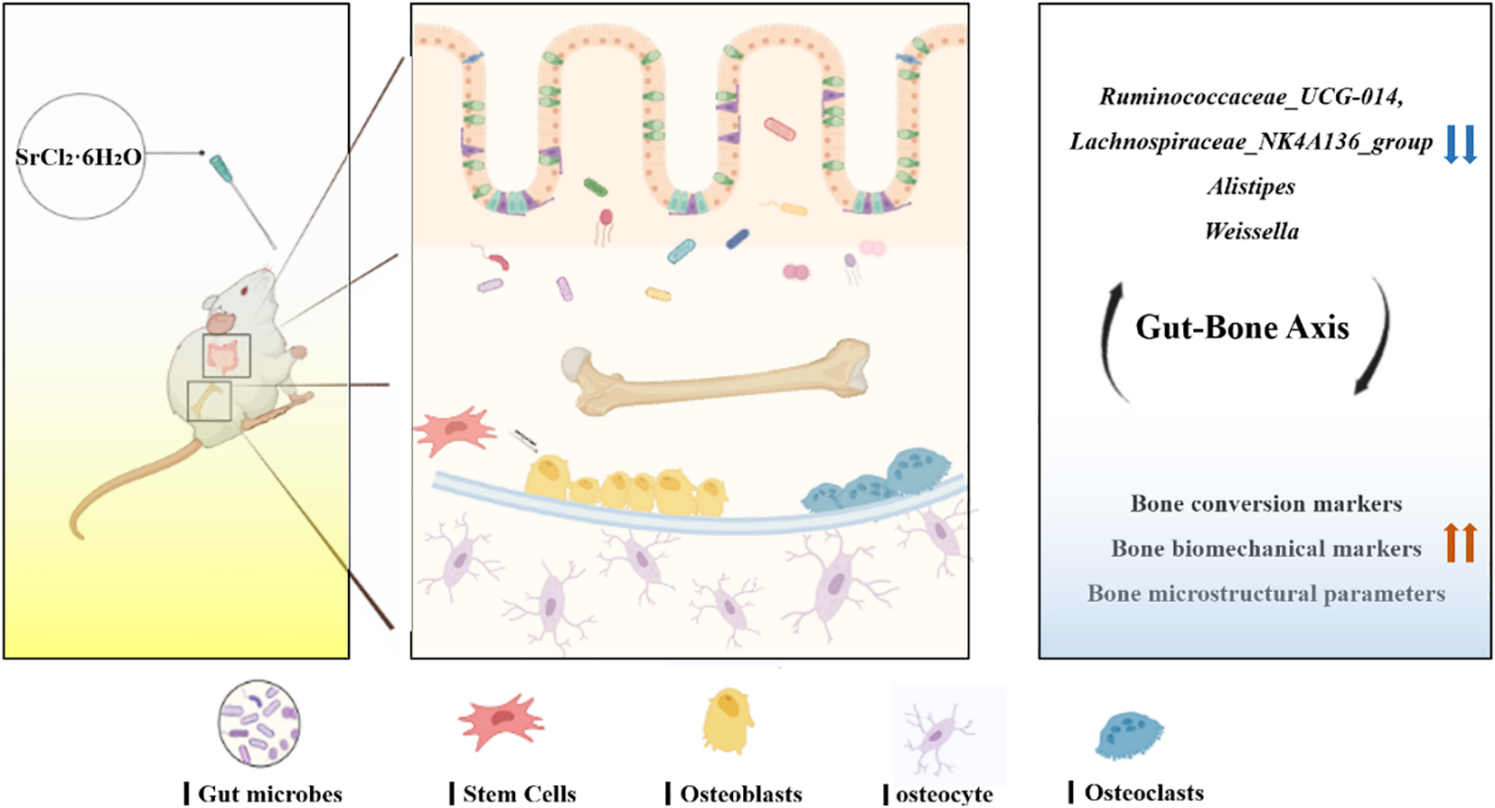
Mechanism of regulation of bone metabolism in the “gut-bone axis” mediated by strontium chloride supplementation via intestinal flora. Strontium treatment may influence the rate of bone turnover, improve bone microstructure and enhance bone biomechanics by modulating the gut microbiota and altering key bacteria such as *Lachnospiraceae_NK4A136_group, Alistipes, Ruminococcaceae_UCG-014* and *Weissella*.

In the present study, Sr exposure was found to elevate bone transformation markers, enhance bone biomechanical properties and improve bone microstructure parameters, while correlating with changes in some specific flora. We speculate that *Ruminococcaceae_UCG-014, Alistipes, Lachnospiraceae_NK4A136_group* and *Weissella* may be potential key bacteria that can regulate bone homeostasis (**Figure 9**). Sr is a key bioactive element that regulates bone turnover and maintains bone balance, which could mitigate bone loss by altering specific gut microbes. It has great potential to circumvent orthopaedic diseases in later life caused by insufficient bone mass. There are still very few approved Sr-rich foods and pharmaceuticals on the market and our research will help to understand the effects of Sr in targeting the gut microbiota to improve bone quality, thus aiding the development and application of Sr-rich functional foods and pharmaceuticals.

## Data availability statement

The data presented in the study are deposited in the NCBI Sequence Read Archive (SRA) database, accession number PRJNA986280.

## Ethics statement

The animal study was approved by The Committee on the Ethics of Animal Experiments of the Chinese Academy of Agriculture Sciences accepted the study’s procedure (permission number: IAS2022-146). The study was conducted in accordance with the local legislation and institutional requirements.

## Author contributions

XX drafted the manuscript. XX and YG performed the experiments and analyzed the data. YG, JW, and NZ revised the manuscript. NZ designed the experiments and had primary responsibility for the final content of the manuscript. All authors contributed to the article and approved the submitted version.

## References

[1] Available at: http://www.nhc.gov.cn/wjw/zcjd/201810/4988546cfa1040db86c1815d3dad7a2b.shtml.

[2] Nonaka K Murata S Nakano H Anami K Shiraiwa K Abiko T. Association of low bone mass with decreased skeletal muscle mass: A cross-sectional study of community-dwelling older women. Healthcare (Basel) (2020) 8(3):343. doi: 10.3390/healthcare8030343 32947889 PMC7551283

[3] Ziemian SN Ayobami OO Rooney AM Kelly NH Holyoak DT Ross FP. Low bone mass resulting from impaired estrogen signaling in bone increases severity of load-induced osteoarthritis in female mice. Bone (2021) 152:116071. doi: 10.1016/j.bone.2021.116071 34171515 PMC8863567

[4] Al Marzooqi AM Al Saleh J. AB0768 prevalence of low bone mass in patient with early inflammatory arthritis presenting to early arthritis clinic. Ann Rheumatic Dis (2016) 75:1167. doi: 10.1136/annrheumdis-2016-eular.2563

[5] Harding AT Weeks BK Watson SL Beck BR. The LIFTMOR-M (Lifting Intervention For Training Muscle and Osteoporosis Rehabilitation for Men) trial: protocol for a semIrandomised controlled trial of supervised targeted exercise to reduce risk of osteoporotic fracture in older men with low bone mass. BMJ Open (2017) 7(6):e014951. doi: 10.1136/bmjopen-2016-014951 PMC5541517 28611110

[6] Burckhardt P Michel C. The peak bone mass concept. Clin Rheumatol (1989) 8 Suppl 2:16–21. doi: 10.1007/BF02207228 2667868

[7] Ahmed I Amarnani R Fisher C. The metabolic crossroad of the adolescent athlete: achieving peak bone mass during athletic development. Br J Sports Med (2022) 56(23):1330–1. doi: 10.1136/bjsports-2022-105685 35902209

[8] Jandhyala SM Talukdar R Subramanyam C Vuyyuru H Sasikala M Nageshwar Reddy D. Role of the normal gut microbiota. World J Gastroenterol (2015) 21(29):8787–803. doi: 10.3748/wjg.v21.i29.8787 PMC4528021 26269668

[9] Blanton LV Charbonneau MR Salih T Barratt MJ Venkatesh S Ilkaveya O. Gut bacteria that prevent growth impairments transmitted by microbiota from malnourished children. Science (2016) 6275(351). doi: 10.1126/science.aad3311 PMC4787260 26912898

[10] Locantore P Del Gatto V Gelli S Paragliola RM Pontecorvi A. The interplay between immune system and microbiota in osteoporosis. Mediators Inflamm (2020) 2020:3686749. doi: 10.1155/2020/3686749 32184701 PMC7061131

[11] Jhong JH Tsai WH Yang LC Chou CH Lee TY Yeh YT. Heat-killed Lacticaseibacillus paracasei GMNL-653 exerts antiosteoporotic effects by restoring the gut microbiota dysbiosis in ovariectomized mice. Front Nutr (2022) 9:804210. doi: 10.3389/fnut.2022.804210 35187034 PMC8856183

[12] Chedid VG Kane SV. Bone health in patients with inflammatory bowel diseases. J Clin Densitom (2020) 23(2):182–9. doi: 10.1016/j.jocd.2019.07.009 31375349

[13] Sjögren K Engdahl C Henning P Lerner UH Tremaroli V Lagerquist MK. The gut microbiota regulates bone mass in mice. J Bone Miner Res (2012) 27(6):1357–67. doi: 10.1002/jbmr.1588 PMC3415623 22407806

[14] Yan J Herzog JW Tsang K Brennan CA Bower MA Garrett WS. Gut microbiota induce IGF-1 and promote bone formation and growth. Proc Natl Acad Sci USA (2016) 113(47):E7554–63. doi: 10.1073/pnas.1607235113 PMC5127374 27821775

[15] Schrooten I Behets GJ Cabrera WE Vercauteren SR Lamberts LV Verberckmoes SC. Dose-dependent effects of strontium on bone of chronic renal failure rats. Kidney Int (2003) 63(3):927–35. doi: 10.1046/j.1523-1755.2003.00809.x 12631073

[16] Wornham DP Hajjawi MO Orriss IR Arnett TR. Strontium potently inhibits mineralisation in bone-forming primary rat osteoblast cultures and reduces numbers of osteoclasts in mouse marrow cultures. Osteoporos Int (2014) 25(10):2477–84. doi: 10.1007/s00198-014-2791-5 PMC4176572 25048011

[17] Li D Chen K Duan L Fu T Li J Mu Z. Strontium ranelate incorporated enzyme-cross-linked gelatin nanoparticle/silk fibroin aerogel for osteogenesis in OVX-induced osteoporosis. ACS Biomater Sci Eng (2019) 5(3):1440–51. doi: 10.1021/acsbiomaterials.8b01298 33405619

[18] Geng Z Sang S Wang S Meng F Li Z Zhu S. Optimizing the strontium content to achieve an ideal osseointegration through balancing apatite-forming ability and osteogenic activity. Biomater Adv (2022) 133:112647. doi: 10.1016/j.msec.2022.112647 35067434

[19] Verma DK Kumari P Kanagaraj S. Engineering aspects of incidence, prevalence, and management of osteoarthritis: A review. Ann BioMed Eng (2022) 50(3):237–52. doi: 10.1007/s10439-022-02913-4 35061132

[20] Shuai CJ Sun H Wu P Gao CD Yang YW Guo W. Biosilicate scaffolds for bone regeneration: influence of introducing SrO. Rsc Adv (2017) 7(35):21749–57. doi: 10.1039/C7RA01606A

[21] Britton RA Irwin R Quach D Schaefer L Zhang J Lee T. Probiotic L. reuteri treatment prevents bone loss in a menopausal ovariectomized mouse model. J Cell Physiol (2014) 229(11):1822–30. doi: 10.1002/jcp.24636 PMC4129456 24677054

[22] Yan J Takakura A Zandi-Nejad K Charles JF. Mechanisms of gut microbiota-mediated bone remodeling. Gut Microbes (2018) 9(1):84–92. doi: 10.1080/19490976.2017.1371893 28961041 PMC5914914

[23] Yan Q Cai L Guo W. New advances in improving bone health based on specific gut microbiota. Front Cell Infect Microbiol (2022) 12:821429. doi: 10.3389/fcimb.2022.821429 35860378 PMC9289272

[24] Aveline P Cesaro A Mazor M M Best T Lespessailles E Toumi H. Cumulative effects of strontium ranelate and impact exercise on bone mass in ovariectomized rats. Int J Mol Sci (2021) 22(6):3040. doi: 10.3390/ijms22063040 33809778 PMC8002366

[25] Xia G Zhao Y Yu Z Tian Y Wang Y Wang S. Phosphorylated peptides from Antarctic Krill (Euphausia superba) prevent estrogen deficiency induced osteoporosis by inhibiting bone resorption in ovariectomized rats. J Agric Food Chem (2015) 63(43):9550–7. doi: 10.1021/acs.jafc.5b04263 26456758

[26] Pu F Chen N Xue SH. Calcium intake, calcium homeostasis and health. Food Sci Hum Wellness (2016) 5(1):8–16. doi: 10.1016/j.fshw.2016.01.001

[27] Khosla S Hofbauer LC. Osteoporosis treatment: recent developments and ongoing challenges. Lancet Diabetes Endocrinol (2017) 5(11):898–907. doi: 10.1016/S2213-8587(17)30188-2 28689769 PMC5798872

[28] Geoffroy V Chappard D Marty C Libouban H Ostertag A Lalande A. Strontium ranelate decreases the incidence of new caudal vertebral fractures in a growing mouse model with spontaneous fractures by improving bone microarchitecture. Osteoporos Int (2011) 22(1):289–97. doi: 10.1007/s00198-010-1193-6 20204596

[29] Aimaiti A Wahafu T Keremu A Yicheng L Li C. Strontium ameliorates glucocorticoid inhibition of osteogenesis via the ERK signaling pathway. Biol Trace Elem Res (2020) 197(2):591–8. doi: 10.1007/s12011-019-02009-6 31832923

[30] Bonjour JP Ammann P Rizzoli R. Importance of preclinical studies in the development of drugs for treatment of osteoporosis: a review related to the 1998 WHO guidelines. Osteoporos Int (1999) 9(5):379–93. doi: 10.1007/s001980050161 10550456

[31] Ammann P Shen V Robin B Mauras Y Bonjour JP Rizzoli R. Strontium ranelate improves bone resistance by increasing bone mass and improving architecture in intact female rats [published correction appears in J Bone Miner Res. J Bone Miner Res (2004) 19(12):2012–20. doi: 10.1359/JBMR.040906 15537445

[32] Boyd SK Szabo E Ammann P. Increased bone strength is associated with improved bone microarchitecture in intact female rats treated with strontium ranelate: a finite element analysis study. Bone (2011) 48(5):1109–16. doi: 10.1016/j.bone.2011.01.004 21276882

[33] Abrahamsen B Grove EL Vestergaard P. Nationwide registry-based analysis of cardiovascular risk factors and adverse outcomes in patients treated with strontium ranelate. Osteoporos Int (2014) 25(2):757–62. doi: 10.1007/s00198-013-2469-4 24322475

[34] Liu J Liu J Liu L Zhang G Zhou A Peng X. The gut microbiota alteration and the key bacteria in Astragalus polysaccharides (APS)-improved osteoporosis. Food Res Int (2020) 138(Pt B):109811. doi: 10.1016/j.foodres.2020.109811 33288186

[35] Mei F Meng K Gu Z Yun Y Zhang W Zhang C. Arecanut (areca catechu l.) seed polyphenol-ameliorated osteoporosis by altering gut microbiome via lyz and the immune system in estrogen-deficient rats. J Agric Food Chem (2021) 69(1):246–58. doi: 10.1021/acs.jafc.0c06671 33382620

[36] Xu Y Wang Y Li H Dai Y Chen D Wang M. Altered fecal microbiota composition in older adults with frailty. Front Cell Infect Microbiol (2021) 11:696186. doi: 10.3389/fcimb.2021.696186 34485176 PMC8415883

[37] Raimondo A Lembo S Di Caprio R Donnarumma G Monfrecola G Balato N. Psoriatic cutaneous inflammation promotes human monocyte differentiation into active osteoclasts, facilitating bone damage. Eur J Immunol (2017) 47(6):1062–74. doi: 10.1002/eji.201646774 28386999

[38] Yang M Yin Y Wang F Zhang H Ma X Yin Y. Supplementation with lycium barbarum polysaccharides reduce obesity in high-fat diet-fed mice by modulation of gut microbiota. Front Microbiol (2021) 12:719967. doi: 10.3389/fmicb.2021.719967 34512598 PMC8427603

[39] Weersma RK Zhernakova A Fu J. Interaction between drugs and the gut microbiome. Gut (2020) 69(8):1510–9. doi: 10.1136/gutjnl-2019-320204 PMC7398478 32409589

